# Malic Enzyme 1 (ME1) Promotes Adiposity and Hepatic Steatosis and Induces Circulating Insulin and Leptin in Obese Female Mice

**DOI:** 10.3390/ijms24076613

**Published:** 2023-04-01

**Authors:** Frank A. Simmen, John Mark P. Pabona, Ahmed Al-Dwairi, Iad Alhallak, Maria Theresa E. Montales, Rosalia C. M. Simmen

**Affiliations:** 1 Department of Physiology & Cell Biology, University of Arkansas for Medical Sciences, Little Rock, AR 72205, USA; simmenfranka@uams.edu (F.A.S.); john.pabona@commonspirit.org (J.M.P.P.); ialhallak@uams.edu (I.A.); mariatheresa.montales@commonspirit.org (M.T.E.M.); 2 The Winthrop P. Rockefeller Cancer Institute, University of Arkansas for Medical Sciences, Little Rock, AR 72205, USA; 3 Department of Physiology and Biochemistry, College of Medicine, Jordan University of Science and Technology, Irbid 22110, Jordan; andwairi7@just.edu.jo

**Keywords:** mouse, Malic Enzyme 1 (ME1), adipose, liver, mammary gland, small intestine, insulin, leptin, adiponectin, TLR9, FFAR3

## Abstract

Malic Enzyme 1 (ME1) supports lipogenesis, cholesterol synthesis, and cellular redox potential by catalyzing the decarboxylation of L-malate to pyruvate, and the concomitant reduction of NADP to NADPH. We examined the contribution of ME1 to the development of obesity by provision of an obesogenic diet to C57BL/6 wild type (WT) and MOD-1 (lack ME1 protein) female mice. Adiposity, serum hormone levels, and adipose, mammary gland, liver, and small intestine gene expression patterns were compared between experimental groups after 10 weeks on a diet. Relative to WT female mice, MOD-1 female mice exhibited lower body weights and less adiposity; decreased concentrations of insulin, leptin, and estrogen; higher concentrations of adiponectin and progesterone; smaller-sized mammary gland adipocytes; and reduced hepatosteatosis. MOD-1 mice had diminished expression of *Lep* gene in abdominal fat; *Lep*, *Pparg*, *Klf9*, and *Acaca* genes in mammary glands; *Pparg* and *Cdkn1a* genes in liver; and *Tlr9* and *Ffar3* genes in the small intestine. By contrast, liver expression of *Cdkn2a* and *Lepr* genes was augmented in MOD-1, relative to WT mice. Results document an integrative role for ME1 in development of female obesity, suggest novel linkages with specific pathways/genes, and further support the therapeutic targeting of ME1 for obesity, diabetes, and fatty liver disease.

## 1. Introduction

The continued rise in global rates of obesity over the last 40 plus years has stimulated much research into the genetic and environmental mechanisms underlying this complex disease [1,2]. Similarly, focused investigations are ongoing on the pathways causal to its associations with type 2 diabetes, metabolic syndrome, hypertension, cardiovascular dysfunctions, and cancers. These studies, so far, have led to the recognition of the prominent role of central nervous system (CNS) pathway genes/proteins that control appetite regulation, leading to the onset and manifestation of obesity [2,3]. Genome-wide association studies (GWAS) have identified more than 1100 independent loci associated with this condition, yet for many of these, direct or indirect and mechanistic contributions to obesity remain uncharacterized [2,4].

We and others have previously reported that the gene encoding Malic Enzyme 1 (ME1) is positively associated with adipose tissue accretion in male mice, who are fed a high-fat diet [5,6]. In particular, we found that mutation of the *Me1* gene, resulting in loss of ME1 protein expression, conferred lower levels of circulating leptin and insulin, as well as reduced adipocyte leptin gene expression and smaller adipocytes within the retroperitoneal adipose tissue depot of male mice [6]. Earlier studies have associated ME1 gene/protein as a possible contributor to type 2 diabetes susceptibility in humans [7] and with lipogenesis and adiposity in domestic animals [8,9,10]. A recent review of ME1’s significant metabolic role in lipogenesis, cholesterol synthesis, and cellular redox potential, in part through its contribution to the cellular cytosolic NADPH/NADP pool, raised its potential as a pro-oncogene [11]. In obese mouse models induced by high-fat/Western diets, up-regulation of *Me1* gene expression was noted in adipose, intestine, and other tissues [12,13,14,15]. However, these studies were limited in scope since the analyses were conducted primarily in male mice and did not link increased *Me1* expression with lipogenic signaling networks.

Leptin is an adipocyte-secreted hormone that signals the status of body fat stores to the appetite-regulating circuits of the brain [3,16]. Declines in circulating leptin signal the physiological response of starvation [3]. Decreased circulating leptin levels are also thought to underpin a significant proportion of cases of polygenic obesity, which has prompted genome-wide searches for physiological regulators of leptin expression [17,18]. Such analyses have, to date, yielded only a small number of loci that significantly affect circulating leptin levels and failed to identify ME1 as a candidate locus.

In view of our previous findings documenting a prominent role for ME1 in circulating leptin and insulin concentrations in obese male mice, and the emerging positive relationships between leptin and hyperinsulinemia with several human cancers, including those of the female breast and uterus [19,20,21,22], we herein extended our studies to female MOD-1 (C57BL/6N background) mice. Since female C57BL/6 mice are relatively resistant to obesity with consumption of a conventional high-fat diet, in contrast to male counterparts, we used a modified diet which substituted soy protein isolate for casein as the sole protein source. This modified diet led to increased adiposity in female wild-type mice. We found that mutation of the *Me1* gene in obese female mice led to significantly lower circulating levels of leptin, insulin, and estrogen and reduced adiposity, which was accompanied by novel, tissue-specific alterations in specific gene expression. Results support the premise that, in a mouse model of obesity, ME1 is an important physiological regulator of crosstalk among multiple signaling pathways, regardless of gender, suggesting its potential value as a targeted therapy for obesity, diabetes, fatty liver disease, and cancer in humans.

## 2. Results

### 2.1. A High-Fat Containing, Soy Protein-Based Diet Is Obesogenic for Female C57BL/6 Mice, and a Mutant Me1 Allele Counters This Phenotype

**Figure 1:**
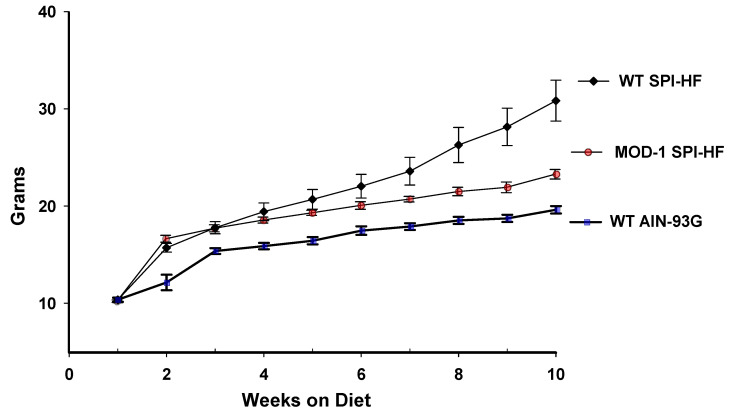
Body weights (mean ± SD) of female mice on each diet (*n* = 10 WT AIN-93G; *n* = 10 WT SPI-HF; *n* = 11 MOD-1 SPI-HF) over a ten week period. By week seven, the three groups significantly differed from each other (*p* < 0.05), and this trend continued until week ten (*p* < 0.05 for each pairwise comparison).

We hypothesized that a high-fat diet containing soy protein isolate (SPI) (designated SPI-HF) would be obesogenic for female C57BL/6 mice. Given that female C57BL/6 mice are not normally responsive to a high-fat diet, relative to male mice, we used this dietary paradigm to evaluate if a mutant Me1 allele (present in the homozygous state in the MOD-1 mouse line) would be protective against developing obesity. The MOD-1 line of mice does not express functional ME1 protein, as monitored by activity [23,24] and Western blot [6]. Accordingly, we put same-aged WT or MOD-1 female mice (postnatal day 21) on three dietary groups as follows: (1) WT mice fed AIN-93G diet (casein-based control diet; non-obese group), (2) WT mice fed SPI-HF diet, and (3) MOD-1 mice fed SPI-HF diet. Body weights at indicated time points over a ten week period were monitored (Figure 1). Mice (WT, AIN-93G) who were fed the control (non-HF diet) showed body weights that were consistently lower than the other groups at the later time points evaluated. At the study’s conclusion, the WT SPI-HF group was significantly heavier that the MOD-1 SPI-HF group.

**Figure 2:**
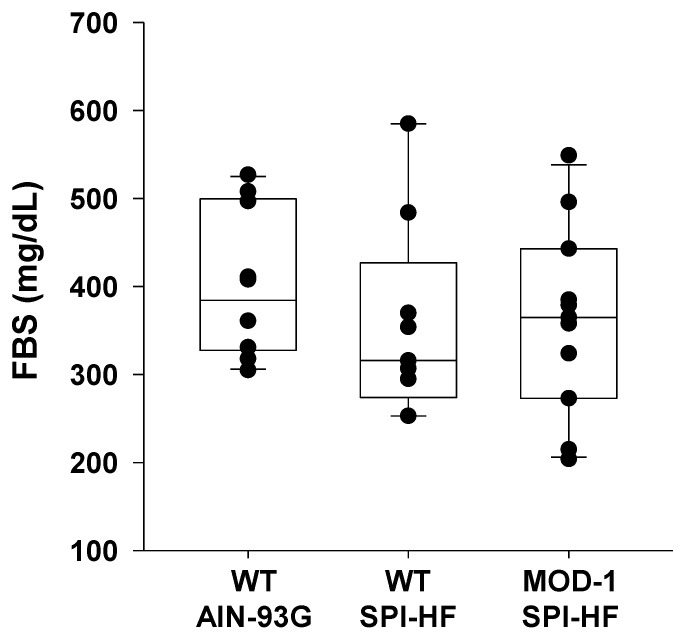
Fasting blood glucose (FBS) levels of experimental mice did not differ at study termination. Groups: *n* = 11 WT AIN-93G; *n* = 9 WT SPI-HF; *n* = 10 MOD-1 SPI-HF. Dots represent values for individual mice. Box plots indicate the upper and lower quartiles, interquartile range, and median (middle line), with whiskers showing maximum and minimum points within 1.5 × the interquartile range. There were no significant group differences or tendencies.

However, the differences in body weights at study termination did not result in differences in fasting blood glucose levels (Figure 2).

**Figure 3:**
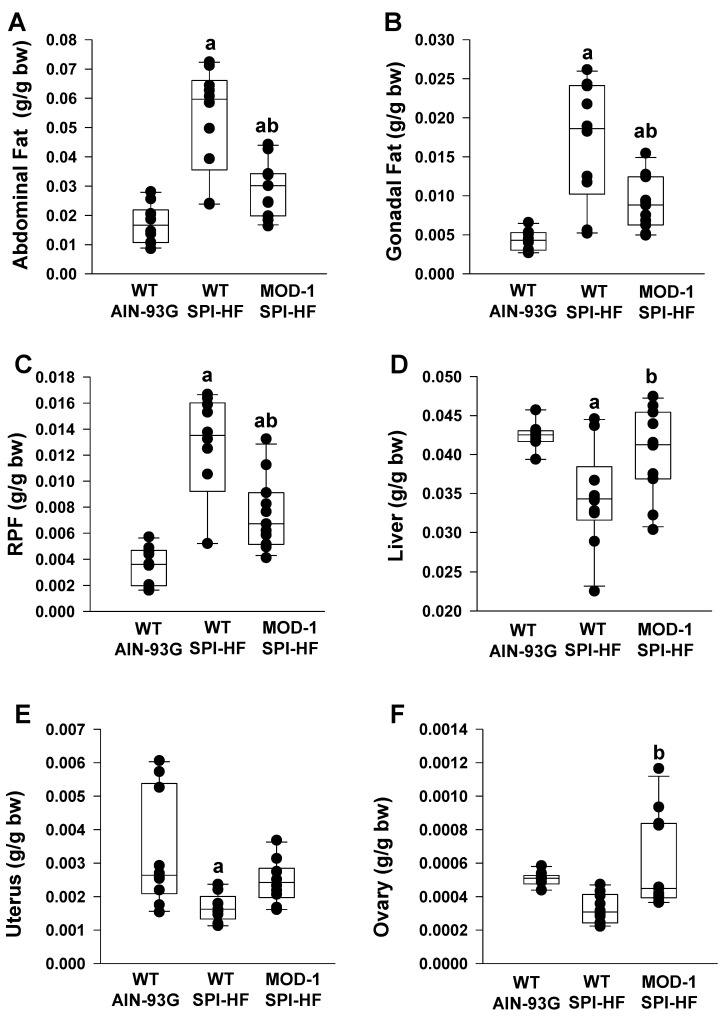
Organ weights (normalized to individual body weight) of female mice fed experimental diets. Mouse groups were *n* = 10 WT AIN-93G; *n* = 10 WT SPI-HF; *n* = 11 MOD-1 SPI-HF. (**A**) Abdominal fat depot, (**B**) gonadal fat depot, (**C**) retroperitoneal fat (RPF) depot, (**D**) liver, (**E**) uterus, and (**F**) ovary. Box plots indicate the upper and lower quartiles, interquartile range, and median (middle line), with whiskers showing maximum and minimum points within 1.5 × the interquartile range. Significant group differences are indicated by a and b (*p* < 0.05); a indicates *p* < 0.05 compared to the WT AIN-93G group and b indicates *p* < 0.05 for WT SPI-HF vs. MOD-1 SPI-HF groups.

Analysis of organ weights, after normalization to individual body weights, demonstrated increased weights of adipose tissue depots (abdominal, gonadal, retroperitoneal) in the WT SPI-HF group compared to the non-obese control group (Figure 3A–C), in agreement with an obese state for the former. The *Me1* mutation conferred partial protection against adiposity (WT SPI-HF vs. MOD-1 SPI-HF) (Figure 3A–C). Interestingly, liver weights followed an inverse pattern to adipose tissue (Figure 3D), with the lowest liver weights demonstrated by the most obese group (WT SPI-HF). Uterine weights for WT SPI-HF group were lower than for the non-obese control group but did not differ from the MOD-1 SPI-HF group (Figure 3E). In contrast, while ovarian weights for WT mice in both diet groups were comparable, the MOD-1 group showed greater ovarian weights than the WT counterparts with a SPI-HF diet (Figure 3F).

### 2.2. Effects of High-Fat Soy Protein Diet and Me1 Mutation on Hormonal Profiles

**Figure 4:**
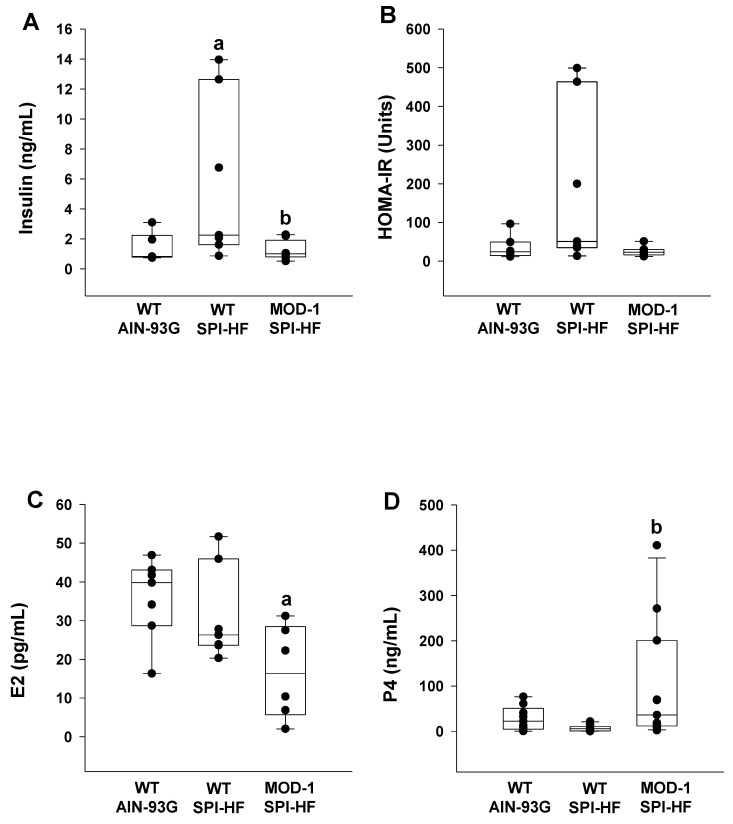
Serum (**A**) insulin, (**B**) HOMA-IR, (**C**) estradiol-17β (E2), and (**D**) progesterone (P4) values for experimental mice (*n* = 7 WT AIN-93G; *n* = 7 WT SPI-HF; *n* = 6 MOD-1 SPI-HF). Box plots indicate the upper and lower quartiles, interquartile range, and median (middle line), with whiskers showing maximum and minimum points within 1.5 × the interquartile range. Significant differences are indicated by a and b; a indicates *p* < 0.05 compared to the WT AIN-93G group and b indicates *p* < 0.05 for WT SPI-HF vs. MOD-1 SPI-HF groups.

Next, we evaluated the serum hormonal status of the experimental female mice to gain insights into altered integrative physiology. The obesogenic diet, relative to the control diet, elevated serum insulin levels in WT mice, but this effect was lost in the mutant group (Figure 4A). Serum Homeostatic Assessment for Insulin Resistance (HOMA-IR) (Figure 4B) tended to follow the measured insulin values (Figure 4A), although statistical differences were not observed. Relative to the control diet, the obesogenic diet had no effect on serum estradiol-17β (E_2_) in WT mice but caused a significant reduction in levels for *Me1* mutant mice relative to WT counterparts (Figure 4C). Conversely, the mutant *Me1* allele caused a significant induction in serum progesterone (P_4_) levels with an obesogenic diet (Figure 4D).

**Figure 5:**
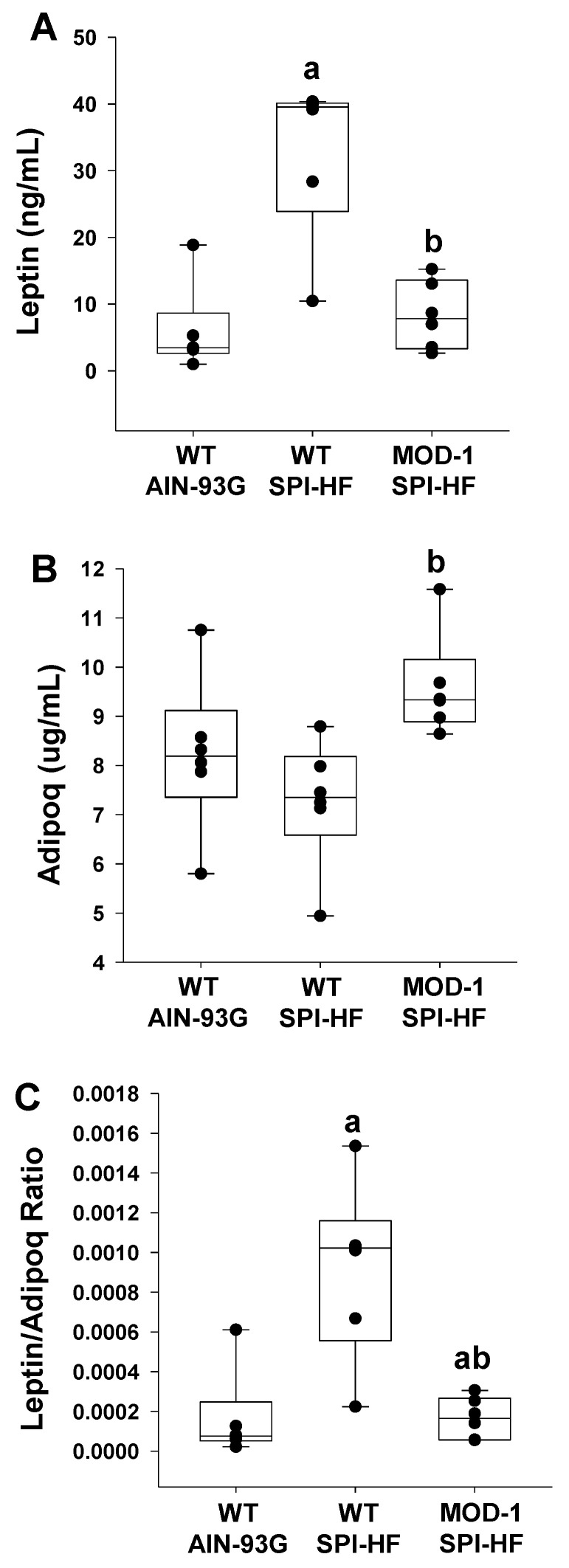
Serum (**A**) leptin, (**B**) adiponectin (Adipoq), and (**C**) leptin/adiponectin ratio for experimental female mice (*n* = 6/group). Box plots indicate the upper and lower quartiles, interquartile range, and median (middle line), with whiskers showing maximum and minimum points within 1.5 × the interquartile range. Significant differences are indicated by a and b; a indicates *p* < 0.05 compared to the WT AIN-93G group and b indicates *p* < 0.05 for WT SPI-HF vs. MOD-1 SPI-HF groups.

The obesogenic diet was associated with elevated serum leptin levels in WT, and this effect of the diet was abrogated with *Me1* mutation (Figure 5A). In contrast, while diet had no effect on serum adiponectin in WT (Figure 5B), *Me1* mutation caused a significant increase in this hormone’s serum levels with an obesogenic diet (Figure 5B). The ratio of serum leptin to adiponectin was elevated by the obesogenic diet in WT, and this effect was partially countered in the *Me1* mutant group (Figure 5C).

### 2.3. Effects of Me1 Mutation on Adipose Tissue Indices

**Figure 6:**
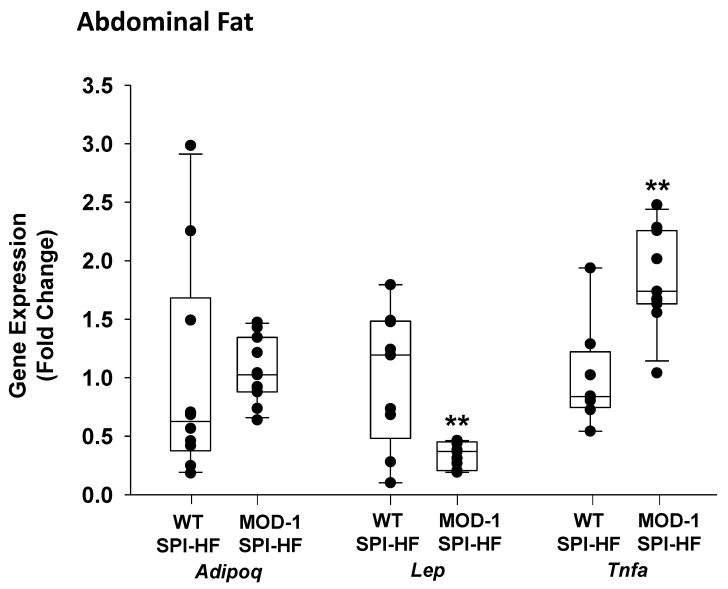
Effects of *Me1* mutation on abdominal fat adiponectin (*Adipoq*), leptin (*Lep*), and tumor necrosis factor α (*TNFα*) mRNA abundance in female mice (*n* = 10 WT; *n* = 11 MOD-1) who were fed a SPI-HF diet. qPCR used *Actb* and *Tbp* as normalizers, followed by application of geNorm, as described previously [25]. Box plots indicate the upper and lower quartiles, interquartile range, and median (middle line), with whiskers showing maximum and minimum points within 1.5 × the interquartile range. ** *p* < 0.01.

Next, we examined abdominal and mammary fat depots for effects of *Me1* mutation. With an obesogenic diet, adiponectin gene expression (i.e., steady-state mRNA abundance) in abdominal fat was unaffected by genotype, while that of leptin was markedly lower in MOD-1 than in WT mice (Figure 6). In contrast, *Tnfa* (TNF-α) expression was significantly elevated in the abdominal fat of MOD-1 mice (Figure 6).

**Figure 7:**
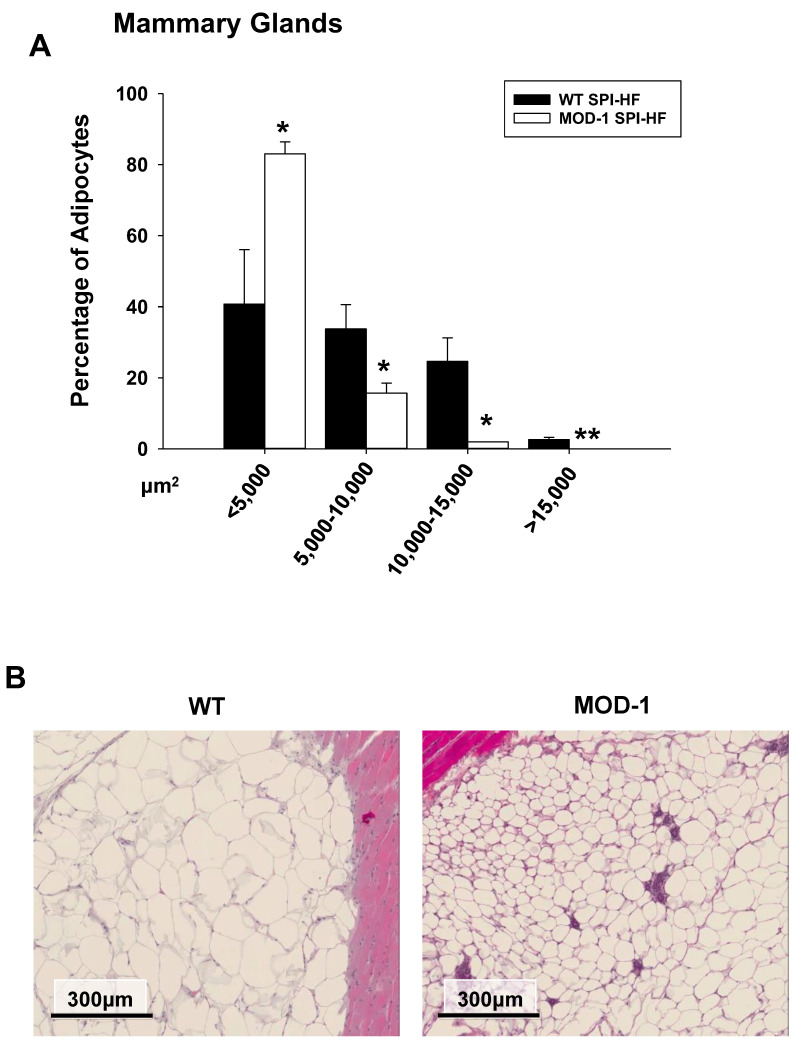
Adipocyte sizes (diameters) in mammary gland fat pads of female WT (*n* = 4) and MOD-1 (*n* = 4) mice who were fed a SPI-HF diet. (**A**) *Me1* mutation led to a shift to smaller sizes of adipocytes; diameters in µm; mean ± SD. * *p* < 0.05, ** *p* < 0.01. (**B**) Representative section (H&E stained) of a mammary gland fat pad from one mouse of each genotype.

Next, we documented the adipocyte size distributions in the mammary fat pads of WT and MOD-1 mice who were fed an obesogenic diet. *Me1* mutation caused a pronounced shift to smaller-sized adipocytes (Figure 7A,B) within this specific adipose tissue depot.

### 2.4. Effects of Me1 Mutation on Select Mammary Gland Genes

**Figure 8:**
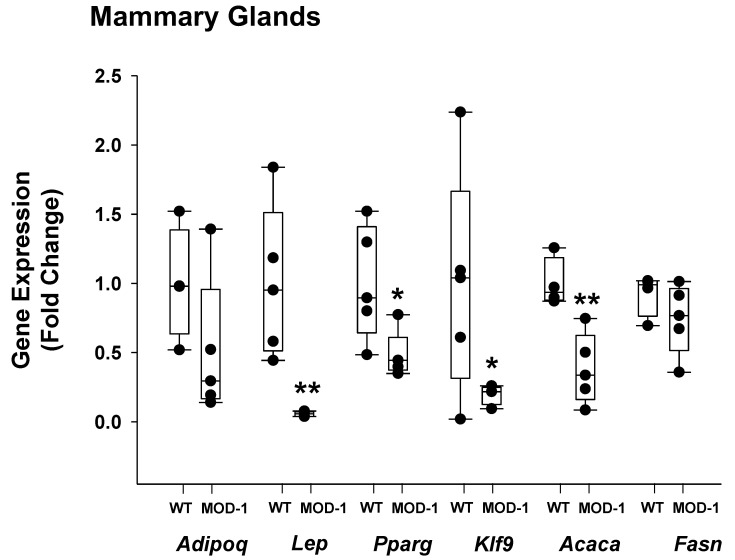
Expression, in mammary gland tissue, of adipogenic/lipogenic genes (female WT and MOD-1 mice who were both fed SPI-HF). qPCR used *Actb*, *Tbp*, and *Ppia* as normalizers, followed by application of geNorm [25]. *n* = 5 mice/group. * *p* < 0.05, ** *p* < 0.01.

Because of the observed adipocyte size differences in the mammary gland fat pad, we determined the relative expression levels of select adipogenic and lipogenic genes in mammary glands (comprised of all cell types including adipocytes) from WT and MOD-1 mice who were fed an obesogenic diet. Our rationale for the analyses is based on the predominant fat cell component of the mammary gland, the tissue’s responsivity to insulin and leptin, and its anatomical correspondence to breast cancer. Mammary gland expression of the genes encoding leptin, PPAR-γ, KLF9, and ACACA were markedly lower in MOD-1 mice compared to WT mice (Figure 8). In contrast, expression of the genes encoding adiponectin (*Adipoq*) and fatty acid synthase (*Fasn*) were unaffected by *Me1* mutation (Figure 8).

### 2.5. Effects of Me1 Mutation on Liver Lipid Content

**Figure 9:**
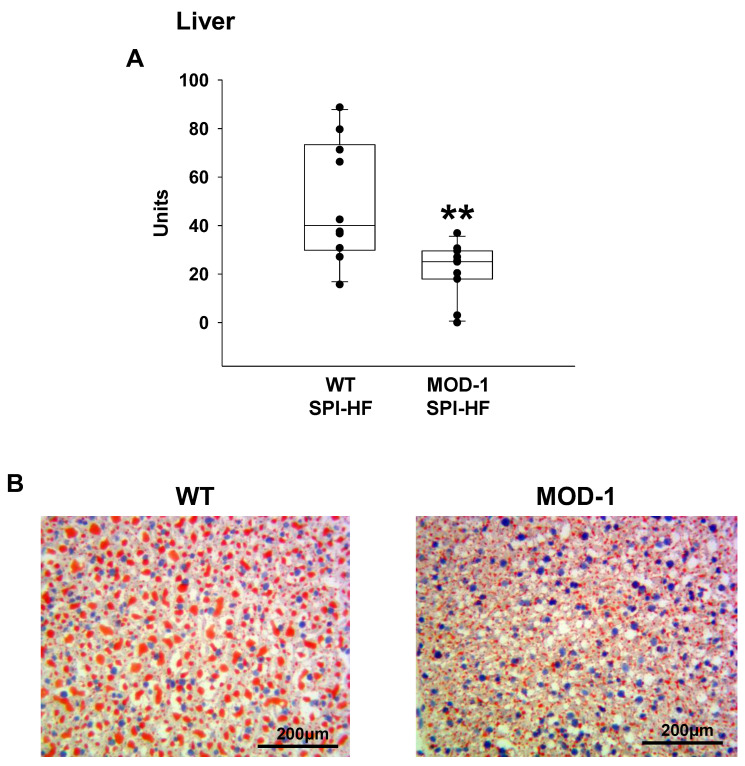
Lipid bodies in mouse livers were visualized and quantified by staining thin sections with Oil Red O, using previously described procedures [6,26]. (**A**) Oil Red O staining intensity was quantified using Aperio ImageScope (Version 12.3); *n* = 10 WT and *n* = 11 MOD-1 mice. ** *p* < 0.01. (**B**) Representative section (Oil Red O-stained) from a liver of one female mouse of each genotype.

In previous work, male MOD-1 mice had less liver steatosis than WT when both groups were fed an HF diet [6]. Therefore, we employed staining with Oil Red O to compare lipid content of livers from HF-diet fed female WT and MOD-1 mice. Remarkably, WT mouse livers exhibited marked macrosteatosis, whereas MOD-1 mouse livers had fewer lipid droplet numbers and reduced droplet sizes (Figure 9).

### 2.6. Effects of Me1 Mutation on Liver Gene Expression Patterns

**Figure 10:**
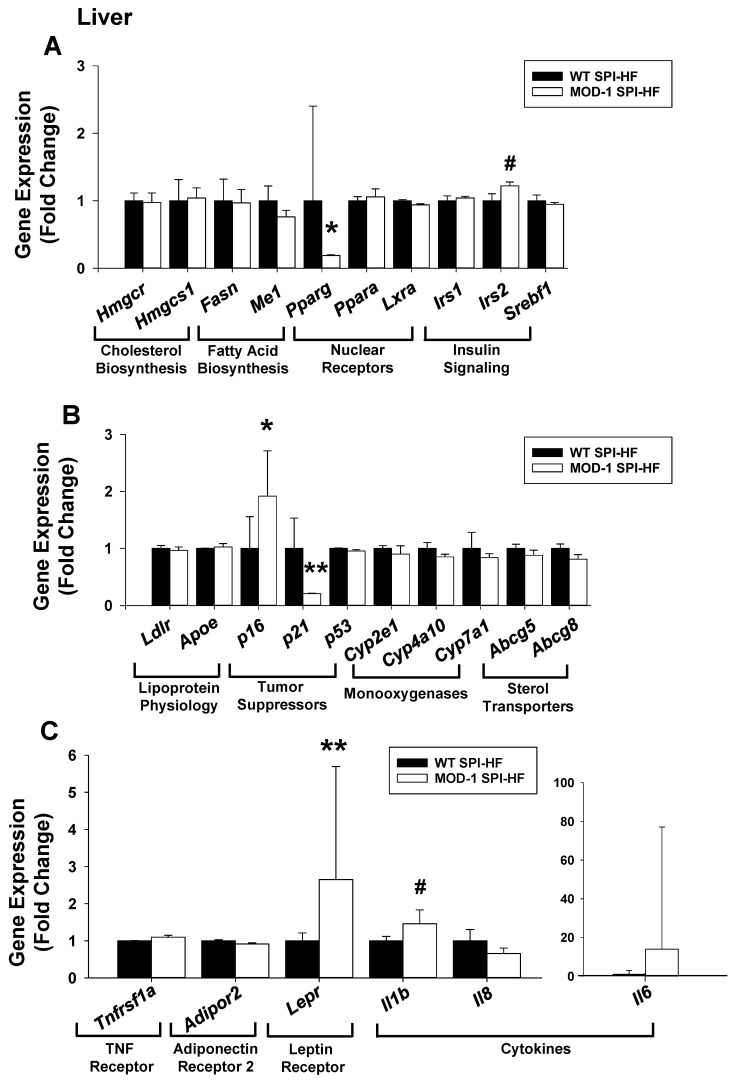
Differential expression of hepatic genes in WT vs. MOD-1 female mice. (**A**–**C**) qPCR of transcripts encoding select proteins in key pathways. qPCR used *Actb* and *Tbp* as normalizers, followed by application of geNorm [25]. Data are mean ± SD for *n* = 10 WT and *n* = 11 MOD-1 mice. * *p* < 0.05, ** *p* < 0.01, # 0.05 < *p* < 0.1.

Our previous work with male mice demonstrated lower hepatic gene expression of Fatty Acid Synthase (FASN) with *Me1* mutation [6]. Here, we employed qPCR to compare liver transcript levels in HF-diet fed WT vs. MOD-1 female mice for a select group of genes whose proteins are involved in cholesterol biosynthesis, lipogenesis, cell cycle regulation, inflammatory signaling, and related pathways (Figure 10). Many of the examined genes were comparably expressed in livers from the two genotypes; however, there were several notable exceptions (Figure 10). Gene transcripts that were significantly greater in female MOD-1 livers included *Cdkn2a* (p16) and *Lepr* (leptin receptor), with *Irs2* and *Il1b* tending (0.05 < *p* < 0.1) in the same direction. In contrast, *Pparg* (PPAR-gamma) and *Cdkn1a* (p21) mRNAs were of lower abundance in MOD-1 than WT livers (Figure 10).

**Figure 11:**
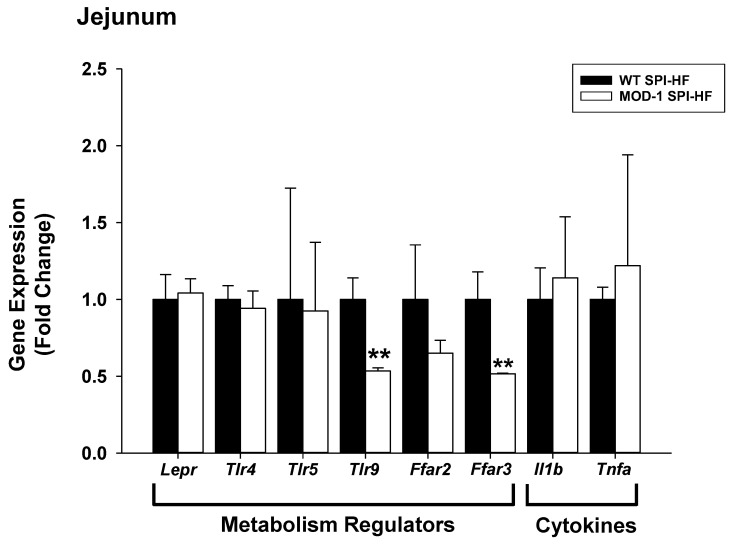
Differences in specific gene expression in jejunums from WT and MOD-1 female mice who were fed an obesogenic diet. Quantification (via qPCR) of transcripts encoding select immunomodulatory and metabolic regulatory proteins. qPCR used *Actb* and *Tbp* as normalizers, followed by application of geNorm [25]. Data are mean ± SD for *n* = 10 WT and *n* = 11 MOD-1 mice. ** *p* < 0.01.

Previous work with male MOD-1 mice, as well as transgenic mice over-expressing ME1 in intestines, pointed to the integrative nature of ME1 expressed in the intestines, liver, and adipose tissues [6,26]. Thus, several metabolic (LEPR, TLRs, FFARs) and immune (cytokines IL-1β, TNF-α) regulatory genes implicated in intestinal barrier function and signaling were evaluated in the small intestines (jejunum) of HF-diet fed WT and MOD-1 females (Figure 11). Of these, only *Tlr9* and *Ffar3* mRNA abundance differed between genotypes and, in both cases, was lower for the MOD-1 mice (Figure 11).

## 3. Discussion

In our previous work, a global *Me1* mutation (resulting in loss of ME1 protein expression) protected male C57BL/6 mice against high fat diet-induced obesity and associated sequelae, including hyperleptinemia, hyperinsulinemia, and hepatosteatosis [6]. In view of possible sexual dimorphism in these characteristics, the present study was conducted to evaluate the contribution of ME1 to the development of the obesogenic phenotype in female counterparts. To induce obesity in female mice, we used a high fat- and soy protein isolate-containing diet, given previous reports that soy protein isolates, as well as pure soy isoflavone genistein, when given orally, are obesogenic/adipogenic in multiple (though not all) rodent models [27,28,29,30,31]. Moreover, increased hepatosteatosis in female, but not male, ob/ob mice was observed with dietary genistein [32]. Estrogen is protective against obesity in female rodents [33,34] and the mechanism of action of genistein in promoting adiposity may be due, in part, to its antagonism of endogenous estrogens at the level of target tissues/cells and estrogen receptors [35,36]. Regardless of the underlying mechanism, use of the SPI-HF diet enabled examination of effects of *Me1* mutation on female mouse obesity and accompanying sequelae. Our findings reported herein extend previous work, that used male *Me1* mutant mice, to the female sex and contribute novel insights on downstream actions of the mutant *Me1* genotype/phenotype on tissue-specific gene expression in the obese state.

It is noteworthy that obese female MOD-1 mice had significantly diminished adipose tissue accretion, lower circulating leptin and insulin concentrations, and higher circulating adiponectin concentrations when compared to obese WT females; except for circulating adiponectin, results were concordant with previous results for male mice [6]. We conclude that ME1 is an adipogenic gene/protein in mice of both sexes and that the effects of ME1 on adipogenesis are best revealed after obesogenic diet consumption. ME1 has a long (and continuing) history as a metabolic enzyme of scientific interest. The ME1 gene is rapidly induced, in tissue-specific fashion, by insulin, thyroid hormone, PPAR ligands, and high-carbohydrate and high-fat diets [12,13,14,15,37,38,39,40]. Interestingly, despite pronounced adipogenic/obesogenic effects in both male and female mice, loss of ME1 protein did not alter glucose levels in either sex. We suggest that the limited experimental duration in our previous [6] and present studies was insufficient to induce the noted hyperglycemia usually associated with a high fat/obesogenic diet.

We found that, in female mice, the group with the greatest adipose tissue weights (i.e., WT SPI-HF) manifested lower liver, uterine, and ovary weights when normalized to individual body weights. In contrast, the liver weights in obese male mice were decreased with *Me1* mutation [6]. These results suggest that ME1 serves as a regulator of metabolic partitioning between tissues and/or of proliferation underlying allometric tissue growth in female mice, at least under the current dietary paradigm. The effect on liver tissue growth/weight appears to be sex-specific, although we cannot rule out potential confounding effects of diet on this parameter.

The liver and adipose tissues play important roles in whole body insulin sensitivity. While measurements of fasting serum glucose and HOMA-IR showed no differences between the experimental groups in our study, serum insulin levels were highest in obese WT females, which were mitigated by *Me1* mutation. This result is consistent with those reported for obese male mice [6], indicating a gender-independent relationship of ME1 and insulin/insulin sensitivity. In this regard, female MOD-1 mice exhibited reduced circulating leptin and increased circulating adiponectin. Both leptin and adiponectin are implicated in whole body insulin sensitivity by virtue of a yin-yang relationship [41]. Thus, it is noteworthy that we observed an altered balance of both hormones in the female MOD-1 mice and in the expected direction for increased insulin sensitivity. In contrast, the increased abundance of TNF-α mRNA in the abdominal fat of obese female MOD-1 mice was unexpected, although it may indicate increased recruitment of macrophages to this tissue. While speculative, we posit that the increased expression of the *Tnfa* gene in abdominal fat may have contributed to localized insulin resistance in this tissue, with a resulting inhibition of insulin action and decreased tissue mass [42].

Parallel evaluation of insulin receptor substrate (IRS) 1 and 2 gene/protein expression have provided additional insights into the association of ME1 with insulin sensitivity. In our previous work with HF-fed male MOD-1 mice, we found that retroperitoneal adipose tissue had significantly greater levels of both *Irs1* and *Irs2* mRNAs [6]. In a subsequent study of transgenic (Tg) mice over-expressing ME1, specifically in the intestines, as well as male MOD-1 mice fed an HF diet, we observed lower levels of *Irs2* (but not *Irs1*) mRNA in Tg jejunum, greater amounts of Phospho-IRS1, and total IRS2 proteins in Tg liver and greater levels of *Irs2*, but not *Irs1*, mRNAs in male MOD-1 jejunum [26]. In the present study, we observed a tendency for increased *Irs2* (but not *Irs1*) transcript levels in livers of obese female MOD-1 mice, relative to obese WT counterparts. Since IRS1 and IRS2 levels and phosphorylation status are known to affect tissue insulin sensitivity [43,44,45], we posit that ME1 effects on tissue insulin sensitivity and circulating insulin levels involve, in part, alterations in tissue-specific expression and actions of IRS1/2 genes and corresponding proteins. The latter raises the question of whether these are direct or indirect effects of ME1. As insulin and insulin signaling pathways are important to normal physiology, as well as pathophysiology, the nature of the ME1–IRS (1/2) network warrants further exploration.

The increased progesterone and decreased estrogen levels in sera of obese MOD-1 mice, relative to WT counterparts, are novel observations for which we currently lack an experimental basis of understanding. The noted differences may indicate altered estrous cyclicity between females of the two genotypes in response to a high-fat diet [46] and/or altered flux/kinetics of the adrenal/ovarian steroidogenic pathways. We noted that ovaries of MOD-1 mice, fed a high-fat diet, were heavier than those of WT mice fed the same diet, which may be attributed to altered follicle and/or corpora lutea numbers/sizes/structures. One report described an important role for ME1 in uterine stromal cell decidualization in response to circulating progesterone during early pregnancy [47]. The latter suggests a functional interplay between serum progesterone and ME1, and the possibility of induction by progesterone of ME1 in the uterine stroma. The female reproductive role(s) of ME1 are relatively unknown, although the collective results herein suggest its potential importance.

Reports concerning the functional role of ME1 in the mammary gland are also currently limited, although lipogenesis and cholesterol biosynthesis are clearly important for mammary gland development and lactogenesis. Our results showed a strong positive association between ME1 and mammary adipose tissue phenotype. We noted a pronounced effect of ME1 to increase mammary adipocyte sizes, as well as mammary expression of *Lep* and *Pparg* mRNAs, the latter encoding a transcription factor (PPAR-γ) that functions as a master regulator of adipogenesis in multiple adipose tissue depots [48]. In addition, ME1 had strong inductive effects on mammary expression of *Klf9*, a pleiotropic transcription factor also implicated in adipogenesis by working in concert with PPAR-γ [49]. ME1 increased gene expression of ACACA, which is highly enriched in lipogenic tissues and constitutes the first and rate-limiting step in fatty acid biosynthesis. Given that mammary adipocytes are an important component of the tumor microenvironment in mammary cancers [50], and since leptin is considered to promote breast cancer [19,20], our results suggest the possible utility of targeting mammary ME1 for breast cancer treatment.

Mouse liver expression of *Pparg* (PPAR-γ), like that for the *Me1* gene, is increased by high-fat diets and mainly occurs within the hepatocyte [14,51]. Since liver-expressed *Pparg* and *Me1* genes are both associated with hepatosteatosis [6,26,51,52,53], the reduced steatosis observed in our female obese MOD-1 mice is likely due, in part, to reduced *Pparg* and absent ME1 expression. The physiological linkages of ME1 and PPAR-γ raise the question of the relevant molecular connection(s). One plausible link may be the transcription factor NFE2-related factor 2 (NRF2), as this nuclear protein functions upstream of both *Me1* and *Pparg* genes in livers of high-fat fed mice [53,54]. Obese female MOD-1 mice showed elevated liver expression of *Lepr*, suggesting the possibility of increased hepatic leptin sensitivity in these mice. While the latter parameter was not measured in the present study, it is worthwhile to note that leptin resistance, along with diminished hepatic leptin receptor gene expression, are characteristics of diet-induced obese mice exhibiting hepatosteatosis [55]. Conversely, the diabetes drug metformin increases mouse hepatic *Lepr* expression while mitigating steatosis [56]. The strong positive associations of hepatic ME1 and steatosis, as we reported in a previous study for male [6], and herein, in female mice, highlight ME1 as a potential drug target to counter fatty liver disease. In addition, the strong associations of ME1 with leptin, PPAR-γ, and TNF-α support significant role(s) for hepatic ME1 in predisposition for insulin resistance and type II diabetes mellitus [57].

The differential effects of ME1 on p21 (decreased) and p16 (increased) expression in liver is a novel, albeit paradoxical, observation. Both p21 and p16 genes/proteins are senescence-associated and serve as inhibitors of the cell cycle. Their divergent regulation (at the level of tissue RNA) in female mouse liver, with an absence of ME1, may be related to the predominant expression of p21, relative to p16 in hepatocytes; in the liver, p16 is found to be predominantly macrophage-derived [58]. In this regard, p16 is reported to be upregulated in liver tissues of obese male mice [59], which is consistent with increased macrophage migration into fatty liver tissue; however, the relevance of this finding in obese female mice has not been examined.

The targeted analysis of a subset of jejunum genes in the context of obesity and ME1 expression revealed novel, positive associations of ME1 with two key mediators of intestinal tissue architecture, inflammation, and metabolism, namely TLR9 and FFAR3. TLR9 agonists trigger Paneth cell secretion of enzymes and antimicrobial peptides in response to the bacterial milieu within the lumen [60]. Moreover, relative to WT counterparts, *Tlr9* KO mice display characteristic small intestine morphology (villus atrophy, crypt disorganization), as well as mis-localization of proliferative progenitor cells to the villus epithelium, when fed a Western diet [61]. In a potential overlap of phenotype with *Tlr9* KO mice, the small intestines of MOD-1 male mice are characterized by shorter villi and a less proliferative phenotype [6]. FFAR3 (previously known as GPR41) serves as a short chain fatty acid (SCFA)-sensing receptor in the intestine and other tissues [62]. While not statistically significant, a numerical decrease in *Ffar2* (previously known as GPR43) mRNA abundance in the MOD1 mouse jejunum was observed. Like FFAR3, FFAR2 serves as a sensing receptor for SCFA [62,63]. Interestingly, both FFAR2 and FFAR3 are implicated in the induction of leptin secretion from adipocytes in response to SCFA [63,64,65,66]. FFAR3 is also expressed in vagal neurons, where it acts in concert with CCK and leptin receptor pathways to control satiety [67]. Our analyses of *Me1* mice revealed striking and novel associations of ME1 with leptin, leptin actions, and intestinal SCFAs, with potential impacts on appetite regulation. Given that ME1, FFAR3, and FFAR2 are all candidate targets for anti-diabetes drugs [63], further elucidation of the networks/pathways that involve these molecules may prove valuable.

In summary, the present results provide support to and further illuminate the involvement of ME1 in the physiological crosstalk of adipose, liver, and small intestine in response to consumption of obesogenic diets. We show that ME1 affects downstream tissue/cell phenotypes and, potentially, cell/tissue dynamics, perhaps directly via altered cytoplasmic NADPH/NADP ratios and/or indirectly by altered ROS signaling and epigenetic regulation [11,68]. With the many tie-ins of ME1 with pathophysiology, the search for small molecule inhibitors of ME1 [69,70] with tissue-selectivity and actions may, in due course, yield useful and multipurpose drugs to treat obesity and associated co-morbidities.

## 4. Materials and Methods

### 4.1. Experimental Animals and Diets

Mouse experiments were conducted following protocols (file #3010 and #3310) approved by the University of Arkansas for Medical Sciences (UAMS) Institutional Animal Care and Use Committee. Mice were housed in the UAMS animal facility under a 12 h light/12 h dark cycle. Female mice at 21 days of age (weaning) were obtained from separate homozygous crosses of wild type (WT; C57BL6/N) and MOD-1 (C57BL6/N) breeder mice [6]. Breeder pairs were fed a standard chow diet. Genotypes were confirmed as described [6]. Weaned female mouse progeny were immediately placed on one of two diets (AIN-93G or SPI-HF; *n* = 10/11 mice/diet/group) for a total of 10 weeks.

Experimental diets were formulated by Harlan Laboratories (Madison, WI) and are described in Table 1. WT females fed an AIN-93G diet (Table 1) comprised a non-obese control group. WT and MOD-1 mice were fed the SPI-HF diet to examine effects of *Me1* mutation on obesity and related parameters. Mice were provided food and water ad libitum and were weighed weekly. After 10 weeks on the diet, mice were weighed and euthanized between 8–11 a.m., ~3 h after food withdrawal.

Liver and retroperitoneal, gonadal, and abdominal (visceral) fat depots were removed by dissection and immediately weighed and frozen. The abdominal cavity was exposed and visceral fat was excised. For gonadal fat isolation, the ovaries were identified and the attached adipose tissue was excised. For retroperitoneal fat depot isolation, which is in a paravertebral position along the border between the posterior abdominal wall and the spinal cord, forceps were used to lift the kidney up and towards midline, followed by dissection of the retroperitoneal fat pad from the posterior peritoneal wall. The right mammary gland #4 of each animal was immediately collected into TRIzol for later RNA extraction [71]. The right mammary gland #3 of each animal was collected for H&E staining [71]. Ovaries and uteri were collected and weighed. The small intestine was divided into thirds and the middle third was taken as the jejunum [6]. Sera was prepared from trunk blood. Tissues and sera were stored at −80 °C until use.

### 4.2. Serum Glucose, Insulin, Leptin, Adiponectin, Estrogen, and Progesterone

Serum glucose levels were determined via glucometer [26]. Serum insulin, leptin, adiponectin, estradiol-17β, and progesterone were measured using commercially available ELISA assays, as previously described by our laboratories [6,26,71]. HOMA-IR was calculated as described [26].

### 4.3. RNA Isolation and Quantitative RT-PCR (qPCR)

RNA was extracted from tissues by use of TRIzol (ThermoFisher, Waltham, MA, USA). Synthesis of cDNA, performance of qPCR, and data normalization and analysis were as described [6,26,71]. Primer sequences are listed in Table 2. The transcripts used for normalization (via geNorm) were β-actin (*Actb*), TATA box-binding protein (*Tbp*), and in one case, Cyclophilin A (*Ppia*).

### 4.4. Histology

Frozen 5 µm sections of livers were stained with Oil Red O, counter-stained with hematoxylin, and subjected to image analysis for lipid droplets using ImageScope (Aperio), as previously described [6,26,72].

Mammary gland (right mammary gland #3; 5 µm) frozen sections were stained with hematoxylin and eosin (H&E) [71]. Five randomly chosen fields from each animal/section underwent analysis for adipocyte size (twenty adipocytes per field) using ImageScope (Aperio Technologies Inc., Vista, CA, USA) [6]. Individual adipocyte areas were calculated from corresponding diameters and were graphically presented as a percentage of adipocytes within a given size range (<5000; 5000–10,000; 10,001–15,000; and >15,000 µm^2^; *n* = 4 mice per group).

### 4.5. Statistical Analysis

Data are presented as the mean ± standard deviation (SD) and were analyzed for statistical differences between two groups using Student’s *t*-test, and, among three groups, one-way analysis of variance (ANOVA), followed by Tukey’s post hoc test was used. Analyses were performed using SigmaStat version 3.5 software (SPSS Inc., Chicago, IL). For Student’s *t*-test results, values are indicated as * *p* < 0.05, ** *p* < 0.001, and # 0.1 > *p* > 0.05. For ANOVA results, *a* indicated *p* < 0.05 compared to the WT AIN-93G group and *b* indicated *p* < 0.05 for WT SPI-HF vs. MOD-1 SPI-HF. Box plots indicate the upper and lower quartiles, interquartile range, and median (middle line), with whiskers showing maximum and minimum points within 1.5 × the interquartile range.

## Figures and Tables

**Table 1:** Experimental diets.

Component	AIN-93G (TD.95092, Harlan) (g/Kg)	SPI-HF (TD.110630, Harlan) (g/Kg)
Isolated Soy Protein	0	210.0
Casein	200.0	0
L-Cystine	3.0	1.2
L-Methionine	0	2.5
Corn starch	397.486	130.0
Maltodextrin	132.0	180.0
Sucrose	100.0	150.0
Lard	0	210.0
Corn oil	70.0	20.0
Cellulose	50.0	28.26
Mineral mix	35.0	50.0
Vitamin mix	10.0	15.0
Choline bitartrate	2.5	3.0
TBHQ, antioxidant	0.014	0.04

SPI-HF: soy protein isolate and high fat. AIN-93G and SPI-HF diets provided 17.2% and 45.2% of total calories from fat and 3.8 and 4.7 Kcal of energy per g of diet, respectively.

**Table 2:** Primers used in qRT-PCR.

Gene	Forward Primer (5′-3′)	Reverse Primer (5′-3′)
***Abcg5***	CCTGAACATTCCAATCCCTTTG	ACGTTTCTATTTCCCGCTCTC
***Abcg8***	GGCAAAGGAACTCAACACAAG	TCCCGGAAGTCATTGGAAATC
***Acaca***	AAGGCTATGTGAAGGATGTGG	CTGTCTGAAGAGGTTAGGGAAG
***Actb***	ACCTTCTACAATGAGCTGCG	CTGGATGGCTACGTACATGG
***Adipoq***	TGTCTGTACGATTGTCAGTGG	GCAGGATTAAGAGGAACAGGAG
***Adipor2***	AGGAAGATGAAGGGTTTATGGG	AATCCGGTAGCACATCGTG
***Apoe***	CAATTGCGAAGATGAAGGCTC	TAATCCCAGAAGCGGTTCAG
***Cyp2e1***	TCACTGGACATCAACTGCC	TGGTCTCTGTTCCTGCAAAG
***Cyp4a10***	CCCAAGTGCCTTTCCTAGATG	GCAAACCATACCCAATCCAAG
***Cyp7a1***	AACGATACACTCTCCACCTTTG	CTGCTTTCATTGCTTCAGGG
***Fasn***	CCCCTCTGTTAATTGGCTCC	TTGTGGAAGTGCAGGTTAGG
***Ffar2***	CTATGTAGCCCAGACCAGC	CACCTGCCAGAACTCCTTG
***Ffar3***	CTTCTTTCTTGGCAATTACTGGC	CCGAAATGGTCAGGTTTAGCAA
***Hmgcr***	AGTCAGTGGGAACTATTGCAC	TTACGTCAACCATAGCTTCCG
***Hmgcs1***	TGTTCTCTTACGGTTCTGGC	AAGTTCTCGAGTCAAGCCTTG
***Il1b***	ACGGACCCCAAAAGATGAAG	TTCTCCACAGCCACAATGAG
***Il6***	CAAAGCCAGAGTCCTTCAGAG	GTCCTTAGCCACTCCTTCTG
***Il8***	TGCTAGTAGAAGGGTGTTGTGCGA	TCCCACACATGTCCTCACCCTAAT
***Irs1***	ATAGCGTAACTGGACATCACAG	GCATCGTACCATCTACTGAAGAG
***Irs2***	GTCCAGGCACTGGAGCTTT	GCTGGTAGCGCTTCACTCTT
***Klf9***	GGCTGTGGGAAAGTCTATGG	AGTGTGGGTCCGGTAGTG
***Ldlr***	ACCCGCCAAGATCAAGAAAG	GCTGGAGATAGAGTGGAGTTTG
***Lep***	AGCCTCACTCTACTCCACAG	CCTCTACATGATTCTTGGGAGC
***Lepr***	ATTTCCTCTTGTGTCCTACTGC	AAGATGCTCAAATGTTTCAGGC
***Lxra***	CCTACGTCTCCATCAACCAC	ACACTTGCTCTGAATGGACG
***Me1***	AGTATCCATGACAAAGGGCAC	ATCCCATTACAGCCAAGGTC
***P16***	GTGCGATATTTGCGTTCCG	TCTGCTCTTGGGATTGGC
***P21***	CAGATCCACAGCGATATCCAG	AGAGACAACGGCACACTTTG
***P53***	ATGTTCCGGGAGCTGAATG	CCCCACTTTCTTGACCATTG
***Ppara***	CATTTCCCTGTTTGTGGCTG	ATCTGGATGGTTGCTCTGC
***Pparg***	TGTTATGGGTGAAACTCTGGG	AGAGCTGATTCCGAAGTTGG
***Ppia***	GCAGACAAAGTTCCAAAGACAG	CATTATGGCGTGTAAAGTCACC
***Srebf1***	CCATCGACTACATCCGCTTC	GCCCTCCATAGACACATCTG
***Tbp***	AAGAAAGGGAGAATCATGGACC	GAGTAAGTCCTGTGCCGTAAG
***Tlr4***	TTCAGAACTTCAGTGGCTGG	TGTTAGTCCAGAGAAACTTCCTG
***Tlr5***	CATCTGTGAGACACCCCTTG	CAGGGAGATATTACCAACACGG
***Tlr9***	AACCGCCACTTCTATAACCAG	GTAAGACAGAGCAAGGCAGG
***Tnfa***	CTTCTGTCTACTGAACTTCGGG	CAGGCTTGTCACTCGAATTTTG
***Tnfrsf1a***	CTCTGCTCTACGAATCACTCTG	CACAGCATACAGAATCGCAAG

## Data Availability

The data reported herein are available upon request from the corresponding author.
